# The Effect of Insulin Resistance and Obesity on Low−Density Lipoprotein Particle Size in Children

**DOI:** 10.4274/jcrpe.v2i2.63

**Published:** 2010-05-02

**Authors:** Mehmet Emre Taşcılar, Tolga Özgen, Murat Cihan, Ayhan Abacı, Ediz Yeşilkaya, İbrahim Eker, Muhiddin Serdar

**Affiliations:** 1 Gulhane Military Medical Academy, Department of Pediatrics, Division of Pediatric Endocrinology, Ankara, Turkey; 2 Bakırköy Maternity and Children Diseases Training and Research Hospital, İstanbul, Turkey; 3 Gulhane Military Medical Academy, Department of Biochemistry, Ankara, Turkey; +90 532 573 20 90+90 212 631 84 46drtolgaozgen@yahoo.comBakırköy Maternity and Children Diseases Training and Research Hospital, Yenimahalle, Bakırköy, İstanbul, Turkey

**Keywords:** Low−density lipoprotein particle size, obesity, insulin resistance

## Abstract

**Objective**: In adults, it was shown that obesity and insulin resistance affect low−density lipoprotein (LDL) particle size and small dense (sd) LDL is associated with cardiovascular diseases. In this study, we investigated the effect of obesity and insulin resistance on LDL particle size.

**Methods**: Twenty−six obese children (13 girls, 13 boys) with a median age of 10.5 years and 27 healthy control subjects (17 girls, 10 boys) with a median age of 11.5 were enrolled in the study.

**Results**: The number of patients with insulin resistance in the obese group was 15 out of 26. In the control group, there was no subject with insulin resistance. Serum triglyceride and very LDL (VLDL) levels were higher and serum high−density lipoprotein levels (HDL) were lower in the obese patients than in the controls. There was no statistical difference in the LDL particle size between the two groups (medians: 26.6 vs. 26.7 nm (p=0.575)). The size of LDL particle was not correlated with body mass index (BMI) standard deviation score (SDS), homeostasis model assessment of insulin resistance (HOMA−IR), or serum lipids.

**Conclusion**: Measurement of LDL particle size as a routine procedure is not necessary in childhood obesity.

**Conflict of interest:**None declared.

## INTRODUCTION

Low−density lipoprotein (LDL) size is heterogeneous in humans and different subtypes of LDL are defined. Among LDL subclasses, two different fractions have been isolated, namely, larger (pattern A) and smaller, more dense particles (pattern B) (1, 2). Genetic and environmental factors may affect LDL particle size (1, 2, 3, 4, 5, 6). The prevalence of pattern B phenotype is 5−10% in women and men younger than 20 years and 30% in older men (1, 2). Generally, expression of the small dense LDL (sd−LDL) phenotype appears in adulthood as a result of genetic, as well as environmental factors. Obesity, dyslipidemia and insulin resistance are accepted as important environmental factors leading to the development of pattern B (6, 7, 9, 10, 11).

Frequency of obesity and obesity−related diseases has increased both in adults and children. Obese individuals carry risks of cardiovascular diseases (CVD) (12). Some risk factors may be present at a young age, and in these patients the lifelong approach for prevention of CVD must be initiated (13, 14). In recent years, it has been shown that small LDL particle size is associated with atherosclerotic coronary artery disease (15, 16). LDL particle size measurement is proposed in adult patients who have a high risk of coronary artery disease (8).

In this study, we aimed to investigate the effects of insulin resistance and obesity on LDL particle size in children and to assess the need for routine measurement of LDL particle size in obese children for the prediction of CVD.

## METHODS

Twenty−six obese children (13 girls, 13 boys) with a median age of 10.5 and 27 healthy control subjects (17 girls, 10 boys) with a median age of 11.5 were enrolled in the study.

Body mass index (BMI) was calculated as weight in kilograms divided by height in square meters. Subjects who had a BMI above the 95^th^ percentile for age and sex or a BMI standard deviation score (SDS) above +2.0 SD were classified as obese (17). Homeostasis model assessment of insulin resistance (HOMA−IR) index (fasting insulin x fasting glucose/22.5) was used for determining insulin resistance (18). Insulin resistance criteria were HOMA−IR>2.5 for prepubertal children and HOMA−IR>4.0 for adolescents (19).

Children with obesity due to syndromes or other known causes were not taken into the study. Control subjects were healthy children. Sixteen patients in the obese group and 15 children in the control group were pubertal.

All blood samples were taken in the morning between 08.00 and 09.00 hours after an over−night fast. The levels of serum insulin, glucose, total cholesterol, triglyceride, high−density lipoprotein (HDL), very low−density lipoprotein (VLDL) and LDL were measured and the serum samples were stored at −80ºC for measuring LDL particle size.

We used polyacrylamide gradient gel electrophoresis to eliminate the interference of fatty acids and devised a simple, precise method of polyacrylamide gradient gel electrophoresis to measure the diameter of sd−LDL in serum. We used apoferritin and thyroglobulin, which have a molecular diameter of 12.2 nm and 17.0 nm, respectively, and standard LDL particles having a diameter of 25.7 and 27.0 nm as calibrators, estimated by measurement of negative staining of electron microscopy. We also included apoferritin as an internal standard for polyacrylamide gradient gel electrophoresis. The only stain used was Coomassie brilliant blue, and it was used for lipoprotein staining. When we used LDL of 25.73 nm in diameter as a quality control specimen, the coefficient of variation of the size measurements obtained by our method was less than 1.2%.

Informed parent consent was taken for each child and this study was approved by the local ethics committee.

**Statistics**

All statistics were performed using the program SPSS 15.0 for Windows. Mann−Whitney U test was used for comparisons of two groups. Spearman’s correlations were used for calculating correlations between parameters.

## RESULTS

The number of patients with insulin resistance in the obese group was 15 out of 26. In the control group, there was no subject with insulin resistance. The value of HOMA−IR was higher in obese patients than in the controls (p<0.001). Serum triglyceride and VLDL levels were higher and serum HDL level was lower in obese patients than in controls (p<0.05). There was no statistical difference in the LDL particle size between the two groups. Clinical and laboratory features of the groups are shown in Table 1.

Serum triglycerides and VLDL were positively, and HDL was negatively correlated with HOMA−IR (Table 2), while LDL particle size was not correlated with any of these values. The size of LDL particles was not correlated with BMI SDS or HOMA−IR. Four subjects, two in obese group and two in the control group, had sd−LDL.

**Table 1:**
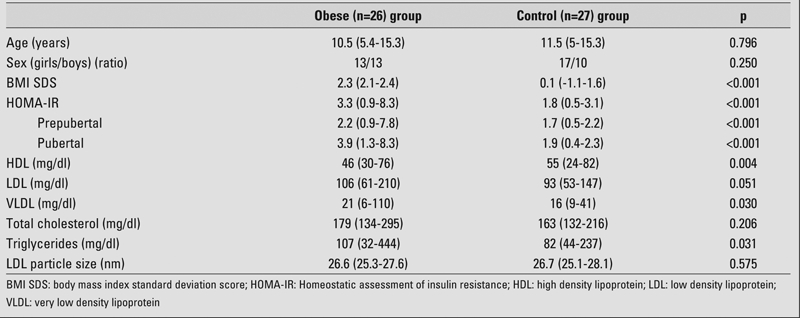
Clinical and laboratory features of the groups (median values and ranges)

**Table 2:**
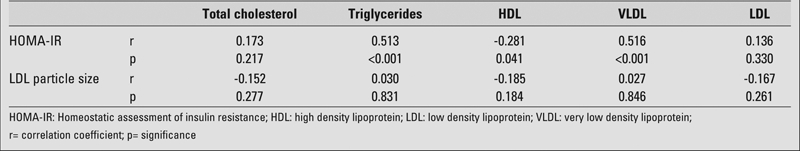
Correlation coefficients between HOMA−IR, LDL particle size and serum lipids

## DISCUSSION

In this study, we investigated LDL particle size in obese children and its relationship with insulin resistance and serum lipid levels. Obesity, the expanding problem of the world, precipitates cardiovascular diseases. Autopsy studies have demonstrated that atherosclerotic process begins in childhood (20, 21). Some risk factors for ischemic heart diseases in the obese population are dyslipidemia, hypertension and insulin resistance (22). In recent years, small LDL particle size has been suggested to be one of the risk factors. Different mechanisms have been proposed for atherogenicity of sd−LDL (23, 24). Björnheden at al (24) have reported that sd−LDL may be taken up easily by arterial tissue because of greater transendothelial transport of smaller particles (24).

It has been reported that the prevalence of sd−LDL was 9.3% in children (25) and 44% in adults (26). Miyashita et al (27) reported that 40% of obese children have sd−LDL. In our study, the prevalence of sd−LDL in the obese group was not as high as that reported by Miyashita’s group. Different factors may play a role in the explanation of our results. Firstly, the genetic make−up of the Turkish population may be different. Genetic factors are important. Previously, the inheritance of sd−LDL was reported as autosomal dominant (28). It has been reported that dominant inheritance has an age−dependent penetration. Our group of subjects was also younger than Miyashita’s group. Shea et al (29) have demonstrated that plasma insulin may modulate lipid levels and particle size in very young children. Rainwanter et al (30) have reported that young men and women had a lower prevalence of small LDL than men and women of an older age.

In adults, it has been demonstrated that serum triglyceride levels are major determinants of LDL particle size (31). In case of hypertriglyceridemia, VLDL transfers its triglycerides to HDL. Triglycerides of this HDL are transferred to the LDL and the cholesterol of LDL is removed from the molecule. This form of LDL with high triglyceride and low cholesterol becomes smaller and denser (32, 33). Obesity is another factor that influences LDL particle size (34). Yoshino et al (35) have reported that LDL particle size increases with weight reduction, although serum lipids do not change significantly.

In our study group, we did not find any relationship between obesity and LDL particle size. Dyslipidemia existed in our children, nevertheless, it did not have any correlation with the pathological clinical and laboratory findings. Although obese children had higher triglycerides and lower HDL than controls, LDL particle size did not have any correlation with these laboratory findings and obesity.

In conclusion, according to our results, it is not necessary to evaluate routinely LDL particle size in obese children. Additional studies with a large number of subjects are needed to evaluate more definitely LDL particle size status, especially in children.
